# Coronary microvascular dysfunction is a hallmark of all subtypes of MINOCA

**DOI:** 10.1007/s00392-023-02294-1

**Published:** 2023-09-02

**Authors:** Andrea Milzi, Rosalia Dettori, Richard Karl Lubberich, Sebastian Reith, Michael Frick, Kathrin Burgmaier, Nikolaus Marx, Mathias Burgmaier

**Affiliations:** 1 Department of Internal Medicine I, University Hospital of the RWTH, Aachen, Germany; 2 Department of Cardiology, Angiology and Electrophysiology, St. Franziskus Hospital, Münster, Germany; 3 Faculty of Applied Healthcare Science, Deggendorf Institute of Technology, Deggendorf, Germany

**Keywords:** MINOCA, Coronary microvascular dysfunction, Coronary physiology, Coronary artery, Disease

## Abstract

**Introduction:**

Myocardial infarction without obstructive coronary artery disease (MINOCA) is a heterogeneous clinical condition presenting with myocardial necrosis not due to an obstruction of a major coronary artery. Recently, a relevant role of coronary microvascular dysfunction (CMD) in the pathogenesis of MINOCA has been suggested; however, data on this are scarce. Particularly, it is unclear if CMD is equally present in all subtypes of MINOCA or differentially identifies one or more of these conditions. Therefore, the aim of this study was to assess CMD in all three coronary vessels of MINOCA patients, relating it with the clinical subtype.

**Methods:**

We retrospectively assessed coronary microvascular function in all three coronary territories by means of angiography-based index of microvascular resistance (aIMR) in 92 patients (64 with working diagnosis of MINOCA, 28 control patients). To further assess the association of CMD with MINOCA subtypes, MINOCA patients were subdivided according to clinical data in coronary cause (*n* = 13), takotsubo (*n* = 13), infiltrative or inflammatory cardiomyopathy (*n* = 9) or unclear (*n* = 29).

**Results:**

Patients with working diagnosis of MINOCA showed a significantly elevated average aIMR compared to control patients (30.5 ± 7.6 vs. 22.1 ± 5.9, *p* < 0.001) as a marker of a relevant CMD; these data were consistent in all vessels. Among MINOCA subtypes, no significant difference in average aIMR could be detected between patients with coronary cause (33.2 ± 6.6), takotsubo cardiomyopathy (29.2 ± 6.9), infiltrative or inflammatory cardiomyopathy (28.1 ± 6.8) or unclear cause (30.6 ± 8.5; *p* = 0.412). Interestingly, aIMR was significantly elevated in the coronary vessel supplying the diseased myocardium compared with other vessels (31.9 ± 11.4 vs. 27.8 ± 8.2, *p* = 0.049).

**Conclusion:**

Coronary microvascular dysfunction is a hallmark of all MINOCA subtypes. This study adds to the pathophysiological understanding of MINOCA and sheds light into the role of CMD in MINOCA.

## Introduction

Myocardial infarction with no obstructive coronary artery disease (MINOCA) represents a form of myocardial infarction (MI) without angiographic evidence of relevant coronary stenoses [1, 2]; MINOCA accounts for up to 5–7% of patients with MIs [3]. In daily practice, several pathologic entities causing myocardial injury are summarized under the “working diagnosis” of MINOCA in the cath laboratory, when no obstructive coronary artery disease is detected in patients with MI [2]. Among others, these mechanisms include plaque activation, coronary embolism, spasm, spontaneous coronary dissection, inflammatory or infiltrative processes; a further peculiar entity sometimes included in MINOCA is represented by takotsubo syndrome [2]. In many of these clinical situations, alterations of the coronary microvascular function are considered to be a key player in determining myocardial injury [4]; nevertheless, due to the difficulty of assessing coronary microvascular function, conclusive studies on the association of coronary microvascular dysfunction (CMD) with MINOCA are lacking. However, recently novel angiography-based methods to assess CMD in all three coronary vessels have emerged [5–7]; these techniques may shed light into the pathophysiology of MINOCA, especially in forms with localized myocardial injury, similarly to what our group showed for patients with localized forms of stable ischemia with no obstructive coronary artery disease (INOCA) [5]. However, data on a localized CMD in patients with MINOCA are scarce.

Therefore, the aim of this study was clarifying the presence and the extent of CMD in all patients with a working diagnosis of MINOCA, also assessing the differences among the different subtypes of this multifaceted clinical condition and different vessels.

## Methods

In this retrospective study, we included 92 patients undergoing coronary angiography at the University Hospital of the RWTH Aachen from May 2019 until August 2022. Sixty-four patients presented with a working diagnosis of MINOCA. The working diagnosis of MINOCA was formulated by the interventional cardiologist at the end of the diagnostic angiography prior to CMR and confirmed ex post by the study team according to symptoms and laboratory values. Working diagnosis of MINOCA was defined as the presence of an elevated high-sensitive troponin level (above the 99th percentile) with a rise/fall, together with signs or symptoms of myocardial ischemia and in the absence of a coronary lesion ≥ 50% in a major epicardial vessel during coronary angiography [1, 2, 8]. Patients with flow-limiting coronary dissections and/or angiographically apparent thrombus burden were excluded from the analysis. As a control group, in order to appreciate differences in microvascular function between patients with and without MINOCA, we included 28 control patients with neither ischemia in stress CMR nor obstructive CAD. In these patients, stress CMR was performed following invasive coronary angiography in order to assess hemodynamic significance of one or more intermediate lesions; in order for patients to be included in this control group, this diagnostic had to be negative with regard to ischemia. These patients have been also part of a previous study [5].

Further inclusion criteria were a sufficient quality of angiographic images to perform measurement of microvascular function according to the methods described below. A cardiac magnetic resonance imaging (CMR) was performed according to local standards. According to the clinical and imaging results, patients with MINOCA working diagnosis were further grouped in 4 subtypes according to the presumed cause: coronary (e.g., embolism, spontaneous coronary dissection, suspect ruptured plaque), takotsubo cardiomyopathy, primary myocardial alterations (e.g., inflammatory or infiltrative pathology or other cardiomyopathies) or unclear. Furthermore, in order to explore local differences in microvascular resistance and their co-localization with diseased myocardium in cMRI, we defined a subgroup of patients which we labeled as “localized disease”; these included all patients demonstrating localized diseased myocardium at cMRI, consistent with the presumed vascularization territory of a single main epicardial vessel.

### Angiography-based assessment of microvascular resistance

**Fig. 1:**
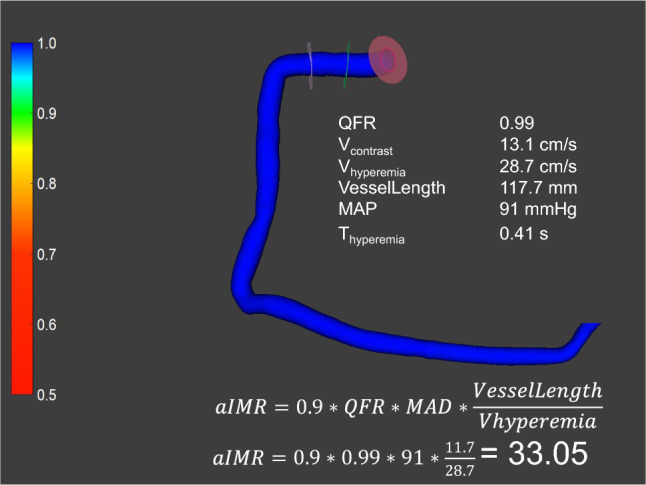
Exemplary analysis of microvascular function from coronary angiography images. As detailed in text, $Vhyperemia=0.1+1.55*Vcontrast-0.93*{Vcontrast}^{2}$

In order to be included in the study, minimum angiography frame rate acquisition needed to be 10 frames/second. Angiographies with lower frame rates were excluded from the analysis. Contrast dye injection was automatically performed with an ACIST injector. Standard settings of the ACIST injectors were 3.0 ml/s, 6 ml, 300 psi for the right and 4.0 ml/s, 8 ml and 300 psi for the left coronary artery. A commercially available software (QAngio XA 3D, Medis Medical Imaging System, Leiden, The Netherlands) was used to calculate quantitative flow ratio [9] and aIMR in all suitable vessels according to previously described protocols [5, 6]. An exemplary measurement is shown in Fig. 1. Mean arterial pressure (MAP) was measured invasively during coronary angiography.

aIMR measurement was performed by an experienced, licensed cardiologist blinded to the clinical presentation of the patient and to the results of other tests (including CMR). As suggested [5], in a non-hyperemic state $$aIMR=0.9*QFR*MAP*\frac{VesselLength}{Vhyperemia}$$, where $$Vhyperemia=0.1+1.55*Vcontrast-0.93*{Vcontrast}^{2}$$

### Statistical analysis

Continuous variables are reported as mean ± standard deviation, dichotomic as count (percentage). Distribution of continuous variables along different groups in the MINOCA population was analyzed by univariate ANOVA; post hoc tests were performed by the LSD test. Distribution of dichotomic variables along different groups in the MINOCA population was assessed by means of the chi-squared test. The comparison of the distribution of aIMR between MINOCA and control patients was performed with t test. Distribution of aIMR in the culprit vs. non-culprit vessel of MINOCA patients with localized alterations in CMR was performed with the paired t test.

All analyses were performed using SPSS v 27.0 (IBM Corp., Armonk, NY, USA). Statistical significance was awarded by *p* < 0.05.

## Results

### Study population

The 64 patients with working diagnosis of MINOCA included in the study were on average 63.6 ± 12.6 years old and in 66% of cases female. The pathologic mechanism of MINOCA was coronary in 13 (20.3%), takotsubo cardiomyopathy in 13 (20.3%), other primary myocardial processes in 9 (14.1%) and unclear in 29 (45.3%) cases. Patients with coronary cause of MINOCA showed significantly elevated high-sensitive troponin and a trend toward elevated CK-MB compared with other causes. Patients with takotsubo cardiomyopathy presented a significantly lower left ventricular ejection fraction (47.2 ± 10.2%) compared with the other groups (*p* = 0.002). Further clinical data stratified for cause of the working diagnosis of MINOCA are reported in Table 1.

**Table 1:** Clinical data of patients with a working diagnosis of MINOCA, stratified for cause

	Cause of “working diagnosis” of MINOCA				*p*
	Coronary (*n* = 13)	Takotsubo (*n* = 13)	Cardiomyopathy (*n* = 9)	Unclear (*n* = 29)	
Male sex (*n*, %)	3 (23.1%)	3 (23.1%)	5 (55.6%)	14 (48.3%)	0.183
Age (years)	58.1 ± 13.7	67.3 ± 11.8	58.5 ± 18.8	64.5 ± 11.8	0.228
Diabetes (*n*, %)	2 (15.4%)	3 (23.1%)	1 (11.1%)	6 (20.7%)	0.88
Hypertension (*n*, %)	6 (46.2%)	5 (38.5%)	4 (44.4%)	20 (69.0%)	0.21
Hyperlipidemia (*n*, %)	3 (23.1%)	2 (15.4%)	4 (44.4%)	10 (34.5%)	0.423
Active smoker (*n*, %)	2 (15.4%)	2 (15.4%)	2 (22.2%)	4 (13.8%)	0.946
BMI (kg/m^2^)	25.6 ± 5.9	23.8 ± 4.0	27.0 ± 5.57	27.5 ± 5.5	0.211
LVEF (%)	57.5 ± 4.9	47.2 ± 10.2	57.5 ± 5.7	57.8 ± 8.6	0.002
Max TropT	541 (547)	492 (490)	116 (113)	116 (161)	0.017
Max CK	350 (396)	194 (122)	182 (177)	176 (226)	0.222
Max CK-MB	54 (64)	33 (15)	26 (8)	30 (20)	0.054

*BMI* body mass index, *LVEF* = left ventricular ejection fraction, *TropT*  high-sensitive troponin T (Roche assay), *CK* = creatine kinase.

Further confirming the absence of obstructive coronary artery disease in the study population, both mean (0.97 ± 0.02, minimum 0.92) and minimal QFR (0.94 ± 0.05, minimum 0.81) over the three major epicardial vessels were in non-ischemic range.

### Microvascular function in patients with working diagnosis of MINOCA

Patients with working diagnosis of MINOCA presented a significantly elevated average aIMR over the three main coronary vessels compared to control patients (30.5 ± 7.6 vs. 22.1 ± 5.9, *p* < 0.001); this difference was consistent in all vessels taken individually (LAD: 36.0 ± 12.1 vs. 21.6 ± 7.7, *p* < 0.001; LCX: 24.1 ± 6.8 vs. 19.1 ± 5.9, *p* < 0.001; and RCA: 31.2 ± 9.3 vs. 26.0 ± 7.5, *p* = 0.016).

**Fig. 2:**
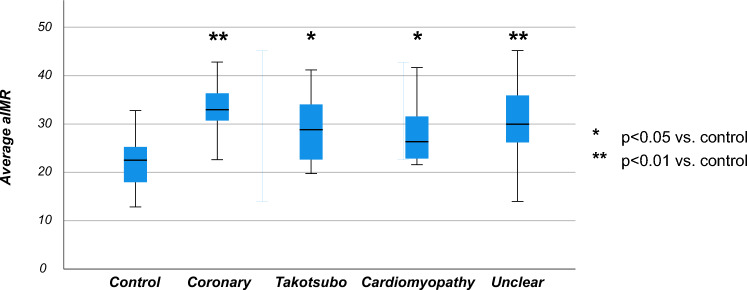
aIMR in the control group and in patients with working diagnosis of MINOCA stratified by cause. Box plot showing the significantly different distributions of aIMR in the control group vs. the different subgroups of patients with a working diagnosis of MINOCA.

No significant difference in microvascular function could be detected in the different clinical entities initially grouped under the working diagnosis of MINOCA (coronary: 33.2 ± 6.6; takotsubo: 29.2 ± 6.9; other cardiomyopathy: 28.1 ± 6.8; and unclear cause: 30.6 ± 8.5; p = 0.412). Nevertheless, all subgroups still presented a significantly elevated aIMR compared to the control group. Data are reported graphically in Fig. 2.

### Single-vessel aIMR co-localizes with diseased myocardium in MINOCA

After demonstrating an overall elevated aIMR in patients with MINOCA compared with control subjects, we analyzed single-vessel coronary microvascular function in patients with localized disease as determined by CMR, in order to investigate if CMD co-localizes with diseased myocardium in MINOCA.

**Fig. 3:**
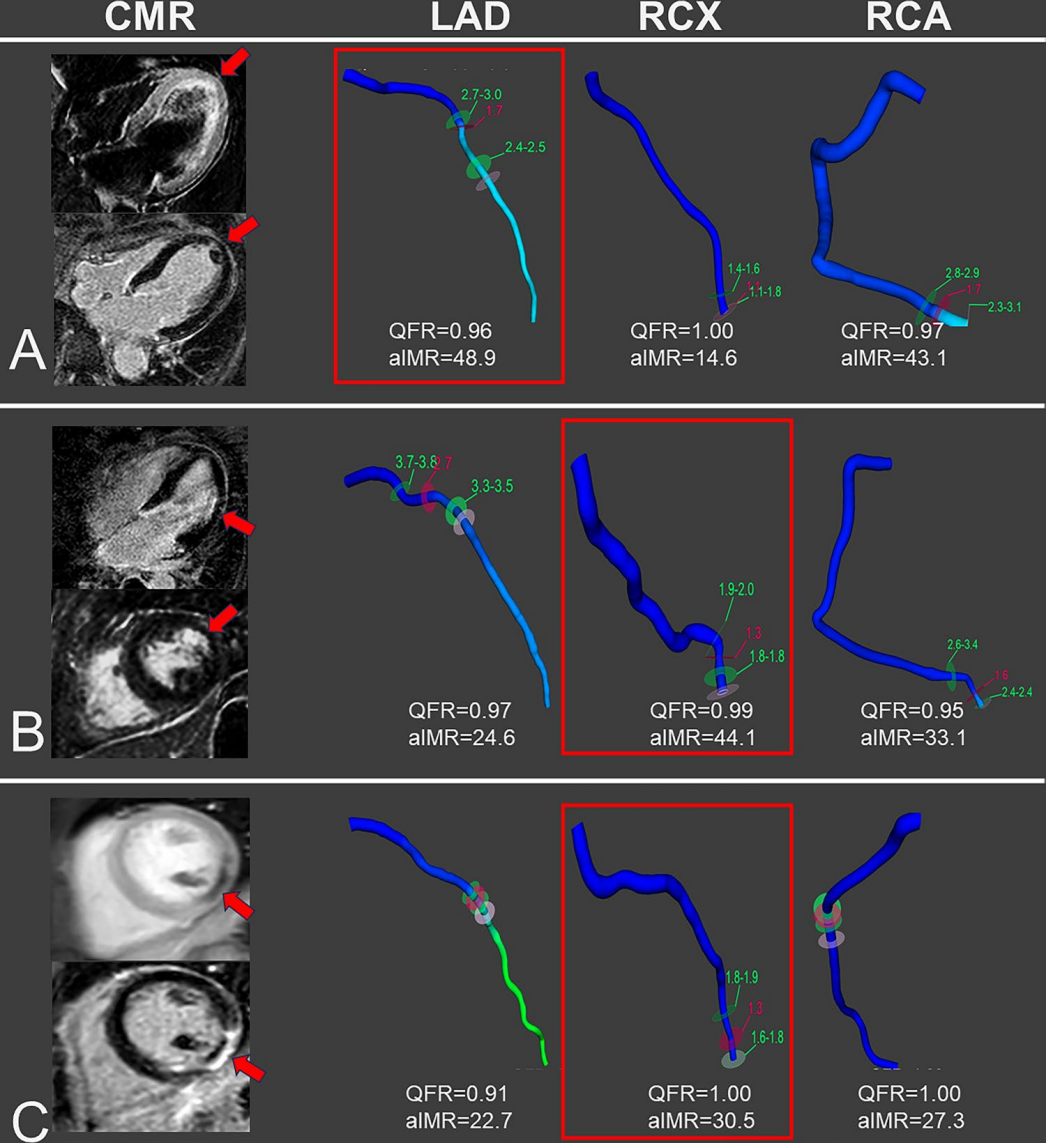
Representative cases of localized MINOCA. For each case, relevant CMR images as well as 3-vessel QFR/aIMR analysis are depicted. The vessel supplying the pathological territory is marked in red. Case A represents a takotsubo cardiomyopathy with typical apical ballooning and LGE. Case B shows a localized infarction of the anterolateral segments, which was not associated with epicardial stenoses. Case C presents LGE and microvascular obstruction following a spontaneous coronary dissection of the RCX, with TIMI-3 flow at the moment of angiogram and with no relevant stenosis

This subgroup included 30 patients, where myocardial alterations were attributable to a single-vessel territory. Vessels supplying pathologic myocardium in CMR showed a significantly elevated aIMR compared with other vessels (31.9 ± 11.4 vs. 27.8 ± 8.2, *p* = 0.049), demonstrating that CMD co-localizes with diseased myocardium in MINOCA. Representative cases of such a co-localization are reported in Fig. 3.

## Discussion

The main finding of our study is the consistent presence of CMD in the coronary vessels of patients with a working diagnosis of MINOCA, as demonstrated by means of an angiography-based measurement. Furthermore, in patients where the MINOCA diagnosis was associated with localized alterations in CMR, CMD co-localizes with diseased myocardium.

MINOCA, i.e., MI without significant obstructive coronary artery disease, is a frequent phenomenon in the cath laboratory and accounts for 5–7% of all MIs [3]. Its pathogenesis is still incompletely understood, but dysregulations in the function of the coronary microvasculature are suggested as a possible mechanism leading to this acute ischemia [4]. The role of CMD in MINOCA is, however, still poorly investigated, as measurements of the microvascular function normally require wire advancement and drug administration, increasing procedure duration and cost; furthermore, this invasive diagnostic modality may bear risks for the patients (especially in the setting of acute coronary syndromes). However, angiography-based measurements have been recently validated as promising tools to assess CMD, without the need for wire advancement or drug administration and therefore with increased patient safety and comfort [5–7]. Therefore, the aim of our study was to use this novel method to retrospectively assess the presence and extent of CMD in patients presenting in the cath laboratory with a working diagnosis of MINOCA.

First, we could demonstrate that patients with a working diagnosis of MINOCA present a significantly higher aIMR compared with control patients. This finding of ours is in line with a previous CMR study, showing a high prevalence of CMD in women with MINOCA [10]. This further supports the hypothesis that CMD is significantly involved in the pathogenesis of MINOCA.

Interestingly, in our study the association of CMD with MINOCA is consistent over the broad spectrum of different causes of MINOCA. MINOCA is a very heterogeneous condition which includes various causes of ischemia, ranging from coronary causes (as embolisms or dissections) to different types of cardiomyopathies. CMR plays a relevant role in differentiating these entities [11] and was therefore used in our study, together with clinical evidence, in order to classify patients with an initial working diagnosis of MINOCA. Still, in spite of this precise subclassification, no significant differences in microvascular function among MINOCA subgroups could be detected in our study. Therefore, CMD may be seen as a hallmark of all different entities included in the working diagnosis of MINOCA. Interpreting this finding needs, however, caution. On the one hand, in some pathophysiological contexts CMD may well be the cause (or at least a contributing cause) of MINOCA: the rarified, chronically dysregulated coronary microvasculature in this case acutely fails to provide a sufficient myocardial blood flow, possibly also as a consequence of an increased demand. Alternatively, the increase in microvascular resistance may be due to an acute hyper-adrenergic state leading to cardiomyocyte death. On the other hand, however, CMD may represent the consequence of all the pathologic processes leading to MINOCA, as, for instance, through compression of the microvessels in infiltrative or hypertrophic cardiomyopathies or through microvascular occlusion following coronary embolization. Moreover, both these options are not mutually exclusive, as a “secondary” CMD may further aggravate ongoing ischemia, as it is, e.g., the case in patients with STEMI [12]. Furthermore, it cannot be excluded that the elevated overall microvascular resistance reported in our study is associated, at least in part, with the acute situation of MINOCA, as an expression of an elevated sympathetic tone inducing increase in microvascular tone. Overall, the elevated aIMR in MINOCA patients probably arises from a combination of all these factors.

Another possible mechanism of ischemia in patients with MINOCA is related to the effects of left ventricular end-diastolic pressure (LVEDP) on coronary perfusion. In fact, coronary perfusion pressure is the difference between the aortic diastolic pressure and LVEDP, so that an increase in LVEDP directly leads to a reduced coronary perfusion pressure. Furthermore, a chronically increased LVEDP results in an increased wall stress on the left ventricle, which in turn causes fibrosis and rarefaction of the coronary microvessels and therefore elicits coronary microvascular dysfunction [13]. Increased LVEDP intervenes therefore on both “functional” and “structural” elements of the coronary microcirculation and simultaneously also increases myocardial oxygen demand. Therefore, LVEDP represents a relevant prognostic factor in both acute [14] and chronic cardiac conditions [15]. Unfortunately, in our study LVEDP has not been systematically registered, so we cannot conclude on its contribution to the ischemia in our patient population. The relationship between an increased LVEDP and CMD in the setting of MINOCA needs to be elucidated in future studies.

Furthermore, we could show that microvascular function is not equally impaired in all coronary vessels during MINOCA. In fact, in the subgroup of patients presenting with a localized myocardial alteration, CMD co-localizes with diseased myocardium in MINOCA. Up to date, similar data had been shown solely in patients with takotsubo cardiomyopathy, a specifical subgroup of patients with a working diagnosis of MINOCA, where anomalies in the apical contractility are associated with an impaired microvascular function in the LAD [16–18]. The finding of our current study also parallels similar findings reported by our group in patients with INOCA, where areas with stress-inducible ischemia but without relevant coronary obstruction expressed a worse microvascular function [5]. Overall, therefore, it is tempting to speculate that microvascular function reveals a very dynamic nature, being subject to heavy local variations; again, in MINOCA patients these variations may be partly the cause of the acute ischemia, but also the consequences of the primary pathologic process causing MINOCA.

Our study presents some limitations. Despite being the first study assessing coronary microvascular function in a wide spectrum of MINOCA subtypes, our sample size remains relatively small, especially with regard to single subtypes; larger, prospective studies are warranted to overcome this limitation. In addition, prospective data based on invasive intracoronary measurements may ultimately be needed. However, for such invasive studies with potential patient risk, prior retrospective studies are of major interest. Furthermore, although previous studies showed an association of CMD with adverse outcomes following MINOCA, we could not conclude on this point due to the lack of prognostic data. Moreover, although our data show a co-localization of locally increased aIMR with diseased segments in MRI, we cannot conclusively assess the correlation between aIMR and perfusion reserve as assessed by MRI itself, as our image acquisition protocol does not allow for such an analysis.

## Conclusion

Coronary microvascular dysfunction, as assessed by means of an angiography-based index of microvascular resistance, is a hallmark of all pathologies presenting with a working diagnosis of MINOCA in the cath laboratory. Interestingly, CMD co-localizes with diseased myocardium in MINOCA, possibly suggesting localized alterations in coronary microvascular function in the pathophysiology of MINOCA.

However, it remains unclear whether the CMD in MINOCA patients represents a causal factor in generating ischemia, or rather an effect of the underlying condition.

## Data Availability

All data will be available upon reasonable request to the corresponding author.
